# Proteomic Analysis of Zika Virus Infected Primary Human Fetal Neural Progenitors Suggests a Role for Doublecortin in the Pathological Consequences of Infection in the Cortex

**DOI:** 10.3389/fmicb.2018.01067

**Published:** 2018-06-05

**Authors:** Xuan Jiang, Xiao Dong, Shi-Hua Li, Yue-Peng Zhou, Simon Rayner, Hui-Min Xia, George F. Gao, Hui Yuan, Ya-Ping Tang, Min-Hua Luo

**Affiliations:** ^1^Joint Center of Translational Precision Medicine, Guangzhou Institute of Pediatrics, Guangzhou Women and Children Medical Center, Guangzhou, China; ^2^Joint Center of Translational Precision Medicine, Wuhan Institute of Virology, Chinese Academy of Sciences, Wuhan, China; ^3^State Key Laboratory of Virology, CAS Center for Excellence in Brain Science and Intelligence Technology, Wuhan Institute of Virology, Chinese Academy of Sciences, Wuhan, China; ^4^CAS Key Laboratory of Pathogenic Microbiology and Immunology, Institute of Microbiology, Chinese Academy of Sciences, Beijing, China; ^5^Department of Medical Genetics, Oslo University Hospital, University of Oslo, Oslo, Norway; ^6^Chinese Center for Disease Control and Prevention, National Institute for Viral Disease Control and Prevention, Beijing, China; ^7^Savaid Medical School, University of Chinese Academy of Sciences, Beijing, China; ^8^Research Network of Immunity and Health, Beijing Institutes of Life Science, Chinese Academy of Sciences, Beijing, China; ^9^Department of Medicine, Medical College, Jianghan University, Wuhan, China

**Keywords:** Zika virus, human fetal neural progenitor cells (NPCs), doublecortin (DCX), cortex structure, proteomic analysis

## Abstract

Zika virus (ZIKV) infection is associated with severe neurological defects in fetuses and newborns, such as microcephaly. However, the underlying mechanisms remain to be elucidated. In this study, proteomic analysis on ZIKV-infected primary human fetal neural progenitor cells (NPCs) revealed that virus infection altered levels of cellular proteins involved in NPC proliferation, differentiation and migration. The transcriptional levels of some of the altered targets were also confirmed by qRT-PCR. Among the altered proteins, doublecortin (DCX) plays an important role in NPC differentiation and migration. Results showed that ZIKV infection downregulated DCX, at both mRNA and protein levels, as early as 1 day post infection (1 dpi), and lasted throughout the virus replication cycle (4 days). The downregulation of DCX was also observed in a ZIKV-infected fetal mouse brain model, which displayed decreased body weight, brain size and weight, as well as defective cortex structure. By screening the ten viral proteins of ZIKV, we found that both the expression of NS4A and NS5 were correlated with the downregulation of both mRNA and protein levels of DCX in NPCs. These data suggest that DCX is modulated following infection of the brain by ZIKV. How these observed changes of DCX expression translate in the pathological consequences of ZIKV infection and if other cellular proteins are equally involved remains to be investigated.

## Introduction

A Zika virus (ZIKV) outbreak has been ongoing in central and south America for the past 2 years, and has raised global concerns with regards to public health (Gulland, 2016; Lazear and Diamond, 2016). ZIKV is a mosquito-borne flavivirus, and its infection is generally either asymptomatic or causes only mild symptoms such as fever, rash, red eyes and joint pain. However, it has been shown to be associated with microcephaly in newborns whose mothers were infected with ZIKV during pregnancy (Musso and Gubler, 2016; Rubin et al., 2016). ZIKV was detected in the placenta and amniotic fluids from the infected women during pregnancy, and also in the blood and brain of congenitally infected fetuses, suggesting a potential for vertical transmission and an association with microcephaly and other birth defects (Calvet et al., 2016; Rubin et al., 2016). ZIKV has been shown to infect neural progenitor cells (NPCs) and inhibits cell proliferation, growth of neurospheres and brain organoids, suggesting that the neuropathology induced by ZIKV might be associated with NPC cell fate (Garcez et al., 2016; Tang et al., 2016). However, the molecular mechanism(s) of ZIKV affecting neurogenesis still remains to be elucidated.

Neurogenesis is a well-organized but complicated process involving the differentiation and maturation of neurons from neural stem cells or NPCs. NPC plays a key role in neurogenesis due to its ability of proliferation and differentiation, and has been extensively utilized for the studies on how ZIKV infection affects neurogenesis. A recent clinical study based on RNA-seq analysis of ZIKV-infected NPCs also showed that NPC intrinsic susceptibility is key to congenital ZIKV syndrome (Caires-Junior et al., 2018), emphasizing the important role of NPCs in ZIKV related neural diseases. It has been shown that ZIKV infection attenuates NPC growth (Tang et al., 2016) and leads to cell-cycle arrest, apoptosis and inhibition of NPC differentiation (Li C. et al., 2016). ZIKV infection also induces mitosis abnormalities and apoptotic cell death of NPCs (Souza et al., 2016). Although there is a study of proteomic analysis on the impacts of ZIKV infection on mosquito C6/36 cells revealing host responses (Xin et al., 2017), the impacts of ZIKV infection on cellular proteins of NPCs had not been investigated on proteomic level. In order to investigate other molecular mechanisms by which ZIKV affects the neurogenesis process, it is necessary to investigate the impacts of ZIKV infection on NPC cellular functions, such as its proliferation, differentiation and migration.

A number of cellular proteins affecting NPC cellular functions, including doublecortin (DCX), have been demonstrated to be essential for neurogenesis (Couillard-Despres et al., 2005; Lie et al., 2005). DCX, mainly expressed in proliferating NPC and immature neurons, is a microtubule-binding protein necessary for migration within the cerebral cortex during neurogenesis (Francis et al., 1999). Defects in DCX expression are responsible for X-linked lissencephaly and subcortical laminar heterotopia (Gleeson et al., 1998), and mutations in the DCX gene cause malformation of the cerebral neocortex (Gleeson et al., 1999). Moreover, depletion of DCX^+^ neuronal precursor cells in the subventricular zone of mice inhibits neurogenesis and neuronal migration (Jin et al., 2010). Human cytomegalovirus, a leading cause of neural development malformation, has been shown to downregulate the mRNA and protein levels of DCX, suggesting a common mechanism by which pathogens can affect neurogenesis (Luo et al., 2010). However, the impact of ZIKV infection on the expression of DCX in NPCs has not been investigated.

ZIKV encodes three structure proteins (C, prM/M, and E) and seven non-structure proteins (NS1, NS2A, NS2B, NS3, NS4A, NS4B, and NS5). Similar to other flaviviruses, each of these viral proteins has multiple functions to affect cellular events (Mukhopadhyay et al., 2005). For instance, ZIKV NS4A and NS4B are reported to suppress Akt-mTOR signaling and induce autophagy (Liang et al., 2016). ZIKV NS5 has been shown to block type I interferon induction and signaling (Hertzog et al., 2018). These findings lead to the investigation on the relationship between viral proteins and the impacts on NPCs, which may further facilitate the unraveling of hidden mechanisms of ZIKV pathogenesis.

In this study, we utilized the primary human fetal NPC and the microinjection of fetal mouse brain to study the mechanisms of how ZIKV infection causes fetal brain development disorders. We found that NPCs were vulnerable to ZIKV infection, that multiple signaling pathways and cellular functions associated with neurogenesis were impaired by the infection, and that ZIKV infection downregulates DCX expression in NPC and mouse fetal brain, which further related to the decreased thickness of cortex layers. Further screening indicated that ZIKV NS4A and NS5 were related to the downregulation of DCX in NPCs, which may contribute to the cortical structure defects caused by ZIKV.

## Materials and methods

### Ethics statement

The isolation of primary NPCs from postmortem fetal embryo tissue was approved by the Institutional Review Board (IRB) (WIVH10201202) of Wuhan Institute of Virology, Chinese Academy of Sciences. The original source of the postmortem fetal embryo tissue was Zhongnan Hospital, Wuhan, China. The need for written or oral consents was waived by IRB. All animals and animal facilities were under the supervision of Wuhan Institute of Virology, Chinese Academy of Sciences. The protocols were approved by the ethics committee and biosafety committee of Wuhan Institute of Virology, Chinese Academy of Sciences.

### Cells and cell culture

Primary human fetal neural progenitor cells (NPCs) were isolated from the brain tissues of postmortem premature neonates and were cultured as neurospheres in uncoated culture dishes or as adherent monolayers in fibronectin-coated dishes as described previously (Luo et al., 2010; Pan et al., 2013). HEK293T cells were cultured as described previously (Li et al., 2015). Vero cells were maintained in Dulbecco's modified Eagle's medium (DMEM, Invitrogen, US) supplemented with 10% fetal bovine serum (FBS, Invitrogen, US) at 37°C with 5% CO_2_.

### Virus and infection

ZIKV (SMGC-1 strain, accession number KX266255) was propagated in Vero cells after being inoculated at a multiplicity of infection (MOI) of 0.01 and supernatants were harvested at 5 days post-infection (dpi). The titers of ZIKV stocks were determined by plaque forming assay on Vero cells as described previously (Yockey et al., 2016).

### Cellular protein extraction and digestion

For quantitative proteomic analysis, NPCs were infected with ZIKV at an MOI of 3 or mock-infected (culture medium was used as mock). At 1 dpi, cells were harvested and washed with prechilled PBS. Cell pellets were resuspended in lysis buffer (4% SDS, 100 mM Tris-HCl, 1 mM DTT, pH 7.6), subjected to sonication and then boiled for 15 min. After centrifugation at 14,000 g for 40 min, the supernatant was quantified with the BCA Protein Assay Kit (Bio-Rad). A total of 200 μg of proteins from each sample were reduced with 100 mM iodoaceamide for 30 min in the dark. The proteins were digested with 4 μg trypsin (Promega) at 37°C overnight, and the resulting peptides were collected as a filtrate. A total of 100 μg peptide mixture from each sample was labeled using TMT reagent according to the manufacturer's instructions (Thermo Fisher Scientific). Pierce high pH reversed-phase fractionation kit (Thermo scientific) was used to fractionate TMT-labeled digest samples into 15 fractions by an increasing acetonitrile step-gradient elution according to manufacturer's instructions.

### LC-MS/MS analysis

For nanoLC-MS/MS analysis, the peptide mixture was loaded onto a reverse phase trap column (Thermo Scientific Acclaim PepMap100, 100 μm × 2 cm, nanoViper C18) connected to a C18-reversed phase analytical column (Thermo Scientific Easy Column, 10 cm long, 75 μm inner diameter, 3μm resin) in buffer A (0.1% Formic acid) and separated with a linear gradient of buffer B (84% acetonitrile and 0.1% Formic acid) at a flow rate of 300 nl/min controlled by IntelliFlow technology. LC-MS/MS analysis was performed on a Q Exactive mass spectrometer (Thermo Scientific) coupled to an Easy nLC nano-flow UHPLC (Proxeon Biosystems, now Thermo Fisher Scientific) for 90 min. The mass spectrometer was operated in positive ion mode. MS data were acquired using a data-dependent top 10 method dynamically choosing the most abundant precursor ions from the survey scan (300–1,800 m/z) for HCD fragmentation. Automatic gain control (AGC) target was set to 3 × 10^6^, and maximum injection time was 10 ms. Dynamic exclusion duration was 40.0 s.

### Protein identification and quantification

Mass spectra were searched using the MASCOT engine (Matrix Science, London, UK; version 2.2) embedded into the Proteome Discoverer 1.4 software package. Search parameters were set as follows: fixed modifications, carbamidomethyl (C), TMT6/10plex (N-term), TMT6/10plex (K); variable modifications, oxidation; peptide mass tolerance, ±20 ppm; fragment mass tolerance, 0.1 Da. The cutoff for the peptide confidence score was 95%, and the protein false-discovery rate (FDR) cutoff was below 1%. The protein ratios were calculated as the median of only unique peptides of the protein. All peptide ratios were normalized by the median protein ratio.

### Bioinformatic analysis

Gene ontology analysis (GO) and Kyoto Encyclopedia of Genes and Genomes (KEGG) pathway annotation and their enrichment analysis were performed as described previously (Chen et al., 2017). Briefly, the protein sequences of differentially expressed proteins were retrieved from the UniProt KB database (Release 2016_10), searched using the NCBI BLAST+ client software (v2.2.28), and loaded into Blast2GO (Version 3.3.5) for GO mapping and annotation. The GO annotation results were plotted by R scripts. The protein sequences that had statistically significant ratios were blasted against the online KEGG database (http://geneontology.org/) and were subsequently mapped to pathways in KEGG4. GO enrichment analysis on the three standard ontologies (biological process, molecular function, and cellular component) and KEGG pathway enrichment analysis were applied and significant differences were identified based on the Fisher's exact test, considering the whole quantified protein annotations as the background dataset. Benjamini-Hochberg correction for multiple testing was then applied to adjust derived *p*-values. Only functional categories and pathways with *p*-values under a threshold of 0.05 were considered as significant.

### Accession number

The mass spectrometry proteomics data have been deposited in the ProteomeXchange Consortium (Deutsch et al., 2017) database via the PRIDE (Vizcaino et al., 2016) partner repository with data set identifier PXD009343.

### Plasmids construction

The coding sequences of ZIKV proteins fused with an N-terminal FLAG-tag were amplified by PCR using the primers with the restriction sites listed in Table 1. The cDNA samples reverse-transcribed from RNA isolated from ZIKV-infected Vero cells were used as templates. Amplified DNA fragments were digested with restriction enzymes and individually ligated into the lentiviral vector pHAGE as described previously (Liu et al., 2017).

**Table 1:** Primers for amplification of the viral and cellular genes.

**Primers**	**Sequence (5′ to 3′)**
**VIRAL GENES**	
C-5′Xho1For	CCGCTCGAGATGGATTACAAGGATGACGACGATAAG AAAAACCCAAAAAAGAAATCCGGAGGATTC
C-3′Xba1Rev	GCTCTAGATTAGACCTCCGCTGCCATAGCTGTG
M-5′Xba1For	GCTCTAGAATGGATTACAAGGATGACGACGATAAGGC TGTGACGCTCCCTTCCCATTC
M-3′BamH1Rev	CGGGATCCTTAGCTGTATGCCGGGGCAATCAG
E-5′Xho1For	CCGCTCGAGATGGATTACAAGGATGACGACGATAAG ATCAGGTGCATAGGAGTCAG
E-3′Xba1Rev	GCTCTAGATTACCCCACATCAGCAGAGAC
NS1-5′Xho1For	CCGCTCGAGATGGATTACAAGGATGACGACGATAAG TGGGGTGCTCGGTGGACTTCTCAAAG
NS1-3′Xba1Rev	GCTCTAGATTAAAGGGAGAAGTGGTCCATGTGATC
NS2A-5′Xho1For	CCGCTCGAGATGGATTACAAGGATGACGACGATAA GGGAGTGCTTGTGATCCTG
NS2A-3′Xba1Rev	GCTCTAGATTAAGGCCAGCTCCGCTTCCCAC TCCTTGTGAG
NS2B-5′Xba1For	GCTCTAGAATGGATTACAAGGATGACGACGATAAG CCTAGCGAAGTACTCACAG
NS2B-3′BamH1Rev	CGGGATCCTTACTCCCCCTTTTTTACTTC
NS3-5′Xho1For	CCGCTCGAGATGGATTACAAGGATGACGACGATAAGA CCACAGATGGAGTGTACAG
NS3-3′Xba1Re	GCTCTAGATTAAGCCGCTCCTCTTTTCCCAG
NS4A-5′Xba1For	GCTCTAGAATGGATTACAAGGATGACGACGATAAGTTT GGAGTGATGGAAGCCCTG
NS4A-3′BamH1Rev	CGGGATCCTTAGGCGGTAATCAAGCCCAGAAG
NS4B-5′Xba1For	GCTCTAGAATGGATTACAAGGATGACGACGATAAG AATGAACTCGGATGGTTGG
NS4B-3′BamH1Rev	CGGGATCCTTAATATTTCACTGGCCTCCTAG
NS5-5′Nhe1Rev	CTAGCTAGCATGGATTACAAGGATGACGACGATAAGA AATATGAGGAGGATGTGAATCTCG
NS5-3′Xho1Rev	CCGCTCGAGTTACAGCACTCCAGGTGTAGAC
**CELLULAR GENES**	
qRT-DCX-For	CCAAGCCTATCATTGTAGTAG
qRT-DCX-Rev	CAGAGGAGAAATCACAGG
qRT-GAPDH-For	GACCCCTTCATTGACCTCAACTA
qRT-GAPDH-Rev	TCCTGGAAGATGGTGATGGG
qRT-EGFR-For	TGGTTATGTCCTCATTGC
qRT-EGFR-Rev	AGATAAGACTGCTAAGGC
qRT-AURKB-For	GATGACTTTGAGATTGGG
qRT-AURKB-Rev	CACGATGAAATGGCTTTTC
qRT-LAMA4-For	CTTACTCATTGGAAGCAC
qRT-LAMA4-Rev	TTGAATCCGGTGGTGTTG
qRT-JUND-For	TCGAGCGCCTCATCATCCAGTC
qRT-JUND-Rev	GTTCTGCTTGTGTAAATCCTC
qRT-NES-For	TGGCACACATGGAGACGTC
qRT-NES-Rev	AACCTCTGTTCCAACGCTG
qRT-CCNB1-For	CAAAACCTTCAGCTACTG
qRT-CCNB1-Rev	TCAGGTTCTGGCTCTGGCACTG

### Lentivirus preparation and transduction

Lentivirus stocks were prepared as described previously (Fu et al., 2015). Briefly, 10 μg of pHAGE plasmid or the constructed lentiviral vector plasmids described above were co-transfected with 10 μg of pMD2G plasmid and 10 μg of pSPAX plasmid via Ca_3_(PO_4_)_2_ precipitation into 1.5 × 10^6^ HEK293T cells. Lentiviruses were harvested at 48 or 72 h post-transfection, cell debris was removed by centrifugation, and the product was frozen at −80°C with 1% DMSO. Stocks were titrated by transducing HEK293T cells with 10-fold serial dilutions in 96-well plates and counting GFP-positive cells at 2 days post-transduction (dpt). NPCs were transduced with lentiviruses at an MOI of 10. Medium was replaced with fresh medium at 3 h post-transduction. Cultures in which over 90% of cells were GFP positive at 5 dpt were harvested for further analysis.

### qRT-PCR

ZIKV-infected NPCs (MOI of 3.0) were harvested at 3, 6, 9, 12, 24, 48, 72, and 96 h post infection (hpi). Cell pellets were processed for RNA extraction using RNAiso reagent (Takara) according to the manufacturer's instructions. Genomic DNA was removed by Dnase I (Thermo), and then 500 ng of total RNA was reverse transcribed using PrimeScript II RTase (Takara) according to the manufacturer's instructions. Real-time qPCR was conducted using a CFX-96 Connect system (Bio-Rad) with iQ SYBR green Supermix (Bio-Rad) using the primers listed in Table 1. All reactions were conducted in triplicate. The relative levels of the target genes were calculated as ratios to the corresponding control following GAPDH normalization. The results were presented as means with standard deviations (SD) from three independent experiments.

### Infection of mouse fetal brain

Specific-pathogen-free ICR mice were purchased from Beijing Vital River Laboratory Animal Technology (licensed by Charles River). 6-week-old ICR mice were mated at embryonic day 0 (E0), and pregnant mice were selected for the ZIKV injection (1 μl of 1 × 10^7^ PFU/ml) into the cerebroventricular space of fetal brains at E13.5. The fetuses were inspected at E18.5 for the body weight, brain size and weight, and either homogenized and lysed for protein detection by immunoblotting or perfused with ice-cold 4% paraformaldehyde (PFA) for protein detection by Immunofluorescence assay (IFA) as described below. All experimental procedures involved were performed according to protocols approved by the Institutional Animal Care and Use Committee of Wuhan institute of Virology, Chinese Academy of Sciences.

### Protein sample preparation and immunoblotting

The mock- or ZIKV-infected NPCs (MOI of 3) were harvested at 1, 2, 3, and 4 dpi. Cell pellets were lysed in radioimmunoprecipitation assay (RIPA) buffer on ice. Cell lysates were prepared as described previously (Liu et al., 2017). The mock- or ZIKV-infected fetal brains were harvested at E18.5 as described above. Fetal brain samples were homogenized with RIPA buffer, followed by sonication. Protein concentrations of cell lysate and fetal brain samples were determined using a BCA protein assay kit (Beyotime, China). Equal amounts of protein of samples were separated on a 10% SDS-PAGE gel and then transferred onto a PVDF membrane (Millie Pore). PVDF membranes were probed with primary antibodies against DCX (Abcam, Cat#ab18723) or GAPDH (Abcam, Cat#A2077) and corresponding horseradish peroxidase (HRP)-conjugated secondary antibodies. The membranes were incubated with SuperSignal West Femto Maximum Sensitivity Substrate (Life Technologies). The chemiluminescent signals were detected using a FluorChem HD2 System (Alpha Innotech) and analyzed densitometrically using ImageJ (National Institutes of Health).

### Immunofluorescence assay (IFA)

NPCs grown on coverslips coated with poly-D-lysine were infected with ZIKV at an MOI of 3 or 0.1, and mock-infected NPCs were used as a control. Coverslips were collected at the indicated time points post-infection and processed for IFA as described previously (Liu et al., 2017). For cryosections, perfused tissues were fixed in 4% PFA, dehydrated in 30% sucrose, and frozen in tissue freezing medium (Leica Biosystems, Germany). Coronal sections (thickness of 40 μm) were used for immunofluorescence staining as described previously (Tang et al., 2015). Mouse monoclonal antibodies for DCX (Abcam, Cat#ab18723), Tbr1 (Abcam, Cat#ab31940), Ctip2 (Abcam, Cat#18465) and anti-ZIKV human serum (Ma et al., 2017) were used as primary antibodies. Secondary antibodies included FITC-anti-mouse-IgG2a (Invitrogen, Cat#11-4210-82), Alexa Fluor 488 goat-anti-human-IgG (Invitrogen, Cat#A-11013) and TRITC-anti-mouse-IgG1 (Southern Biotechnology, Cat#1070-03). Nuclei were counterstained with DAPI (Life Technologies). Images were obtained using a two-photon microscopy with the UltraVIEW VoX 3D Live Cell Imaging System (Perkin Elmer).

### Statistical analysis

The immunoblotting and immunofluorescence images shown are representative of at least three independent experiments. The chemiluminescent signals of the immunoblotting images were densitometrically quantitated using ImageJ (National Institutes of Health). The relative levels of the target proteins were calculated as ratios to the corresponding control following GAPDH normalization. The values are presented below the corresponding blot. The bar graphs were generated by using the GraphPad Prism software package based on the values from three independent experiments, and relative protein levels are represented as average ± standard deviation (SD). Differences were considered statistically significant when *P* < 0.05 based on a Student's *t*-tests analysis. ^*^P < 0.05; ^**^P < 0.01; ^***^P < 0.001.

## Results

### Human NPCs are permissive to ZIKV infection

**Figure 1:**
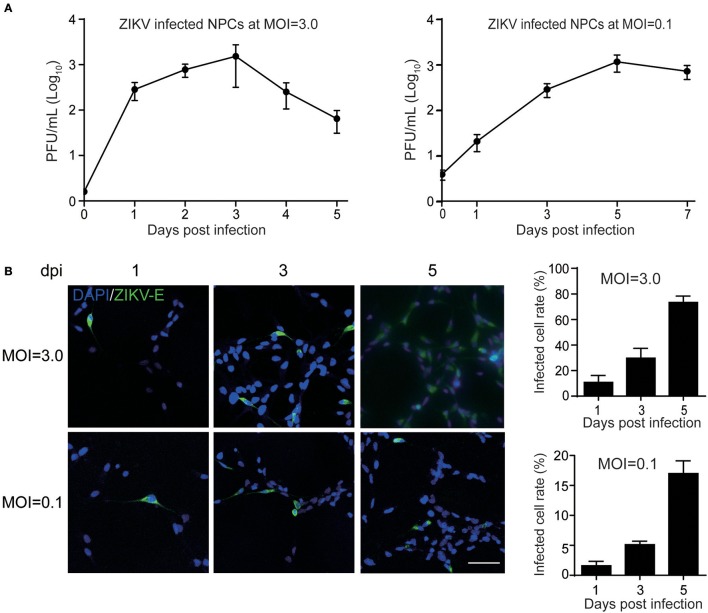
Human neural progenitor cells (NPCs) are permissive to ZIKV infection. NPCs were infected with ZIKV at an MOI of 3.0 and 0.1, respectively, for the detection of virus titers in the supernatant and the infected cell rates. **(A)** ZIKV growth curves. NPCs were infected with ZIKV at an MOI of 3.0 and 0.1, respectively. Supernatant samples were collected at the indicated time points. Virus titers in supernatant samples were determined by plaque forming assay in triplicate. **(B)** ZIKV E protein positive cell rates. NPCs on coverslips were infected with ZIKV at an MOI of 3.0 and 0.1, respectively. Coverslips were collected at the indicated time points. The expression of ZIKV E was stained with anti-ZIKV E protein antibody by IFA. The ZIKV-infected cell rates were determined by counting the ZIKV E protein positive cells vs. total cells stained with DAPI per 500 μm^2^ in 3 different images. Scale bar, 200 μm.

The primary human NPCs used in this study were isolated from two cases at early gestation. NPCs retained a typical identity in terms of expressed NPC markers, such as Nestin, SOX2, GFAP and DCX as described previously (Luo et al., 2008). When NPCs were infected with ZIKV at a high MOI of 3, infectious ZIKV particles in the supernatant increased rapidly and peaked at 3 dpi with a maximal titer of 1 × 10^3^ PFU/ml. At 4 dpi, the virus titer started to decrease when a clear cytopathic effect of cell death appeared with dead cells detached from the culture surface. Moreover, when infected human NPCs were infected with ZIKV at a low MOI of 0.1, the titer of ZIKV in the supernatant initially reached to 30 PFU/ml at 1 dpi, and increased to 1 × 10^3^ PFU/ml at 5 dpi, indicating that similar yield of infectious viral particles can still be reached when infected at a low MOI (Figure 1A). To further monitor ZIKV infection, expression of the ZIKV E gene in NPCs was detected by IFA. The E protein was detected in NPCs upon both low and high MOI infections. The infected cell rates were analyzed according to the E protein signal using ImageJ. At 5 dpi, over 70% of cells were E protein positive when infected with MOI of 3, and less than 20% cells were E protein positive when infected with an MOI of 0.3 (Figure 1B). In addition, the death of ZIKV-infected NPCs was clearly observed and the dead cells detached from the growth surface at 4 dpi, particularly at an infection with an MOI of 3. These data indicated that primary human NPCs were permissive and vulnerable to ZIKV infection.

### ZIKV infection alters multiple pathways and cellular functions of human NPCs

**Figure 2:**
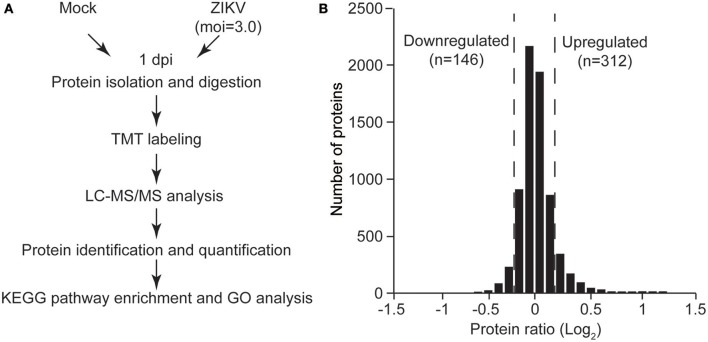
Quantitative proteomic analysis of ZIKV-infected NPCs. **(A)** Schematic of quantitative proteomic analysis of ZIKV-infected NPCs. Briefly, NPCs were infected with ZIKV at an MOI of 3 or mock infected, and were harvested at 1 dpi for protein isolation and digestion. After TMT labeling, the digested peptides were subjected to LC-MS/MS analysis. Proteins were identified and quantified via bioinformatic analysis. KEGG pathway enrichment and GO analysis were performed on differentially regulated proteins. **(B)** Gaussian distribution of measured protein ratios. The proteins with ratios higher than 1.2-fold or lower than 0.8-fold were considered to be differentially regulated.

Having observed the permissibility of primary human to ZIKV infection and their subsequent death, we next sought to clarify the associated molecular mechanisms. Thus, the cellular proteins regulated by ZIKV infection in NPCs were further investigated. TMT-based quantitative proteomic analysis on ZIKV-infected NPCs was performed with mock-infected NPCs as control. Since the virus replication reached a high level at 1 dpi with an MOI of 3.0 (Figure 1A), this same MOI was used in the proteomic analysis and samples were harvested at 1 dpi. A schematic of this process is shown in Figure 2A. A total of 7,039 cellular proteins were identified with 99% confidence at the peptide and protein levels and the cutoff for differentially regulated cellular proteins was set as greater than 1.2-fold or lower than 0.8-fold compared to the mock-infected group as described previously (Chen et al., 2017) (see the complete protein list in Supplementary Table 1). Using this criterion, 312 upregulated cellular proteins and 146 downregulated proteins were identified (Figure 2B).

**Figure 3:**
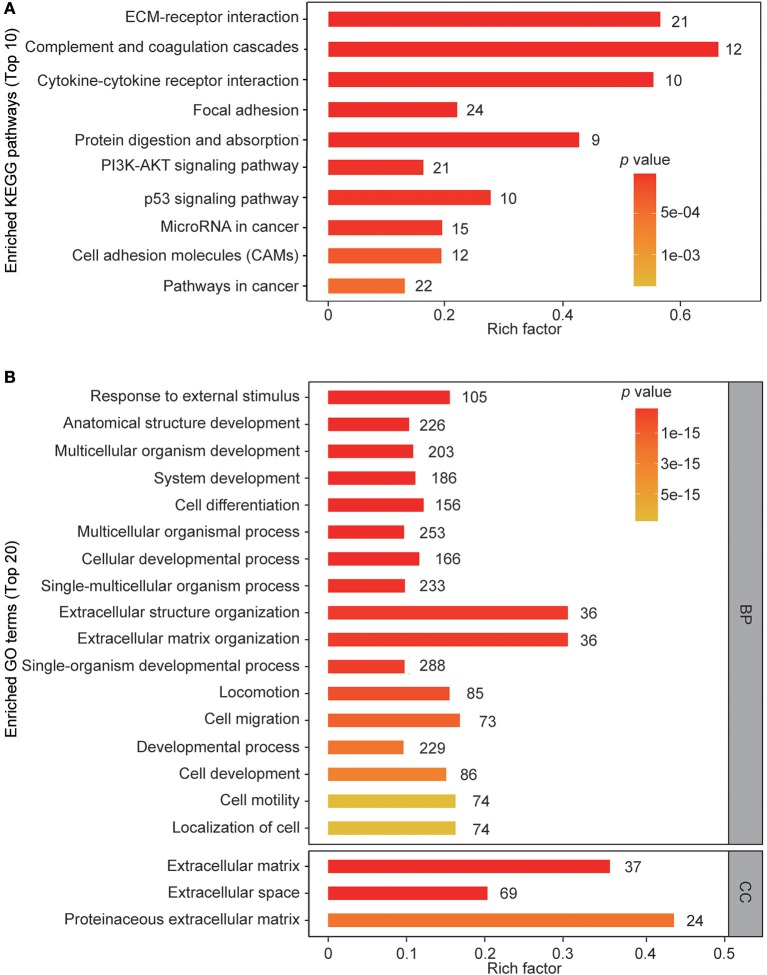
KEGG pathway enrichment and GO terms enrichment of differentially expressed proteins in ZIKV-infected NPCs. KEGG pathway enrichment analysis **(A)** and GO terms enrichment analysis **(B)** were applied based on the Fisher' exact test, considering the entire quantified protein annotations as the background dataset. Benjamini-Hochberg correction for multiple testing was further applied to adjust derived *p*-values. Only functional categories and pathways with *p*-values under a threshold of 0.05 were considered as significant. Numbers of quantified proteins in each enriched pathway or GO term are indicated to the right of the bars.

To gain insights into the biological functions of these differentially regulated cellular proteins, pathway enrichment analysis and gene ontology enrichment analysis were performed. The top 10 enriched KEGG pathways are shown in Figure 3A. The results showed that multiple differentially regulated proteins were enriched in several receptor-ligand interaction pathways (such as “ECM-receptor interaction” and “cytokine-cytokine receptor interaction”) and signaling pathways (such as the “PI3K-AKT signaling pathway” and the “p53 signaling pathway”), indicating that some members of these pathways are significantly affected by ZIKV infection. For GO analysis, the differently regulated cellular proteins were classified according to the three primary GO categories, biological process, cellular component and molecular function. The top 20 enriched GO terms were part of the biological process and cellular component ontologies (Figure 3B). It was found that multiple terms involved in development and differentiation were enriched, including “anatomical structure development,” “system development,” “cell differentiation,” and “cellular developmental process.” These data indicate that ZIKV infection impacts multiple cellular processes and functional pathways related to development and differentiation, which implies an alteration of NPC fate.

To better understand the impact of ZIKV infection on cellular proteins involved in NPC cell fate, annotations of all regulated proteins were examined manually. Representative cellular proteins involved in maintaining NPC status were classified into five groups (Table 2): cell signaling; cell cycle; secreted proteins; transcription; and structural proteins. Many of these proteins have been shown to be related to cellular functions of neural cells, and are found to play roles in cell signaling processes, such as DCX, EGFR, and GAP43, indicating the alteration of multiple cell signaling pathways in NPC by ZIKV infection. Meanwhile, several factors involved in transcription and transcriptional regulation were differently regulated during ZIKV infection, such as FoxG1, JUND, and NFKB1, which play roles in neurogenesis and neuronal maturation. Multiple cellular structural proteins specific to neural cells including GFAP, NES, and NEFL were differentially regulated as well, suggesting the modulation of cell structure and morphology. In addition, two collagen proteins (COL7A1 and COL1A1) and one adhesion protein (LAMA4) had upregulated protein levels, indicating that there may be changes in the adhesion characteristics of ZIKV-infected NPCs.

**Table 2:** Cellular proteins associated with NPC cell fate altered by ZIKV.

**Functional class**	**Symbol**	**Full name**	**Regulation**	**Fold change**
Cell signaling	DCX	Neuronal migration protein doublecortin	Down	0.77
	EGFR	Receptor protein-tyrosine kinase	Up	1.24
	GAP43	Neuromodulin	Up	1.29
	NLGN3	Neuroligin-3	Down	0.79
	NRP2	Neuropilin-2	Up	1.73
	NTM	Neurotrimin	Up	1.21
	OPTN	Optineurin	Up	1.26
	TENM3	Teneurin-3	Up	1.33
Cell cycle	AURKB	Aurora kinase B	Up	1.20
	CCNB1	G2/mitotic-specific cyclin-B1	Up	1.20
	CCPG1	Cell cycle progression protein 1	Up	1.20
	CDC50A	Cell cycle control protein 50A	Up	1.31
	CDCA7L	Cell division cycle associated 7-like	Down	0.75
Secreted	COL7A1	Collagen alpha-1 (VII) chain	Up	1.93
	COL1A1	Collagen alpha-1(I) chain	Up	1.90
	LAMA4	Laminin subunit alpha-4	Up	2.17
	RELN	Reelin	Up	2.05
Transcription	CDK19	Cyclin-dependent kinase 19	Down	0.77
	FOXG1	Forkhead box protein G1	Down	0.76
	JUND	Transcription factor jun-D	Up	1.69
	NFKB1	Nuclear factor NF-kappa-B p105	Up	1.50
	p53	Cellular tumor antigen p53	Up	1.22
	SOX9	Sex determining region Y-box 9	Down	0.75
Structural	CCNDBP1	Cyclin-D1-binding protein 1	Up	1.30
	GFAP	Glial fibrillary acidic protein	Up	1.23
	NEFL	Neurofilament triplet L protein	Up	1.21
	NES	Nestin	Up	1.24
	SEPT4	Septin-4	Up	1.29
	SEPT7	Septin-7	Down	0.71

**Figure 4:**
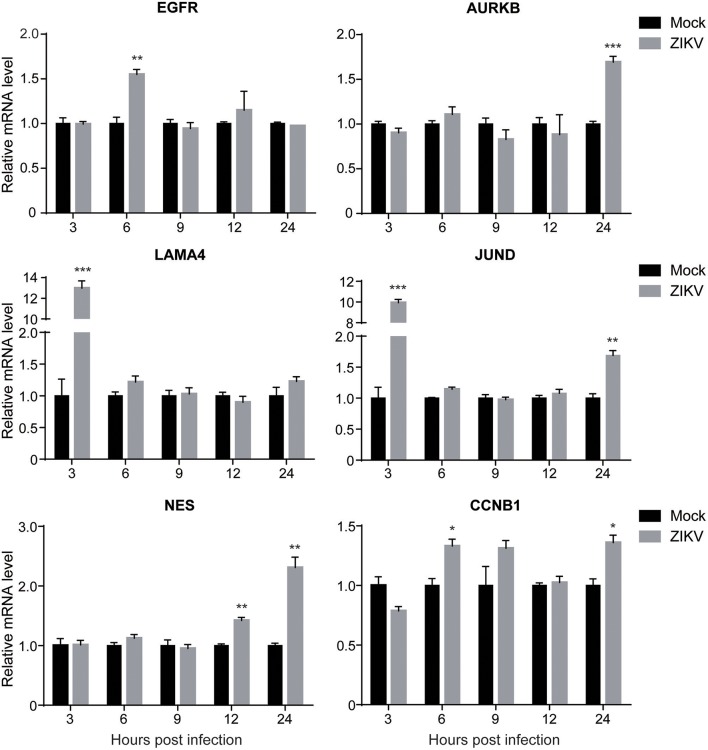
The transcriptional levels of target genes in ZIKV-infected NPCs. NPC monolayers were infected with ZIKV at an MOI of 3.0 or mock infected and RNA was prepared at 3, 6, 9, 12, and 24 hpi. Expression levels of EGFR, AURKB, LAMA4, JUND, NES and CCNB1 were analyzed by qRT-PCR, normalized to GAPDH, and expressed as relative mRNA levels to mock-infected NPCs. Each experiment was conducted in triplicate. Results shown represent the means from three independent experiments. Data were analyzed by one-way ANOVA; ^*^p < 0.05; ^**^p < 0.01; ^***^p < 0.005.

To validate the expression levels of the altered cellular proteins identified by proteomic analysis, the mRNA levels of selected cellular genes corresponding to the proteins in Table 2 (EGFR, AURKB, LAMA4, JUND, NES, and CCNB1) were analyzed by qRT-PCR. The selection criteria include: (1) similar expression levels in three independent groups; (2) fold changes above 1.2; (3) functions in different categories as shown in Table 2. The mRNA of ZIKV- or mock-infected NPCs at MOI of 3.0 at 3, 6, 9, 12, and 24 hpi was isolated and analyzed. In general, the expression levels of these genes were consistent with the proteomic data (Figure 4). The data showed that the expression level of EGFR increased to about 1.5-fold at 6 hpi, while remained the same to mock afterwards. The expression level of AURKB increased to about 1.5-fold at 24 hpi in ZIKV-infected NPCs. The expression levels of LAMA4 and JUND increased to above 10-fold at 3 hpi, and then remained the same to mock afterwards. The expression level of NES started to increase at 12 hpi, and further increased to over 2-fold at 24 hpi. The expression level of CCNB1 increased to 1.3-fold at 6 and 24 hpi. These data validated the protein levels of these genes in proteomic analysis.

### DCX is downregulated at both mRNA and protein levels by ZIKV infection

**Figure 5:**
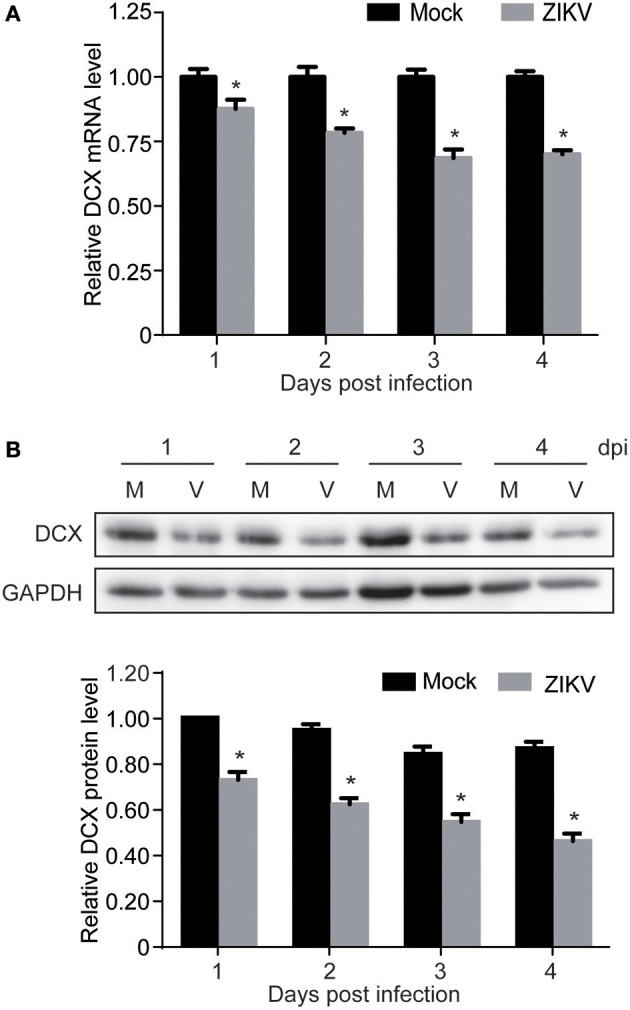
ZIKV infection downregulates DCX in NPCs. NPCs were ZIKV- (V) or mock-infected (M) at an MOI of 3. Infected cells were harvested at the indicated times for DCX mRNA and protein levels analyses. **(A)** DCX mRNA level. Total RNA was extracted from ZIKV- or mock-infected NPCs, reverse-transcribed and DCX mRNA level was assessed by qRT-PCR. Relative DCX mRNA levels in ZIKV-infected NPCs at each time point were normalized to GAPDH mRNA levels and expressed as fold changes relative to mock-infected NPCs. Data were presented as the mean ± SEM from 3 independent experiments. **(B)** DCX protein level. Cell lysates were prepared from infected NPCs. Protein levels were determined by immunoblotting assay. GAPDH was served as a loading control. The DCX levels were quantified by densitometric analysis using ImageJ software and normalized to mock-infected cells at each time point. Data were presented as the mean with SEM of 3 independent experiments. ^*^p < 0.05.

Proteomic analysis showed that DCX, one of the important NPC markers, was downregulated by ZIKV infection at 1 dpi (0.77-fold comparing to mock). This prompted us to further investigate both mRNA and protein levels of DCX throughout a longer infection time course. The qRT-PCR analysis of DCX mRNA levels revealed that the expression level of the DCX gene was decreased at 1 dpi, and continuously decreased to about 70% by 4 dpi (Figure 5A). Similarly, the protein level of DCX was decreased at 1 dpi, and continuously decreased to about 40% compared to the mock-infected NPCs by 4 dpi (Figure 5B). These data suggested that ZIKV infection significantly inhibited expression of DCX at both mRNA and protein levels, implying a downstream influence on the migration process of NPCs during neuronal maturation.

### ZIKV infection causes developmental defects in embryonic mouse model

**Figure 6:**
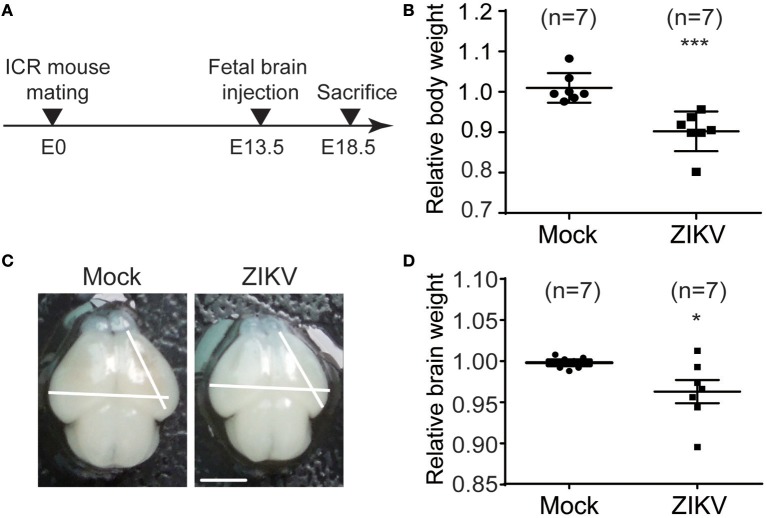
ZIKV infection decreases the body weight, brain size and weight of mouse fetuses. **(A)** Schematic depiction of a ZIKV-infected fetal mouse brain model. ICR mice were mated, and pregnant mice were selected. Fetuses were intracranial injected with ZIKV (104 PFU) or mock-infected at E13.5. Mice were sacrificed at E18.5 and fetuses were harvested for measurements of body weight, brain size and weight. **(B)** Relative body weight. The body weights of mock- and ZIKV-infected fetuses at E18.5 were measured. Data are representative of at least 3 independent experiments with more than 1 pregnant female dam per experiment. The n for each group is indicated above each bar. ^***^p < 0.0001. **(C)** Image of brains. Fetal brains from mock- and ZIKV-infected fetal littermates at E18.5. Scale bar, 5 mm. **(D)** Relative brain weight. Fetal brains of mock- and ZIKV-infected fetuses at E18.5 were measured. Data were collected from at least 3 independent experiments with more than 1 pregnant female dam per experiment. The n for each group is indicated above each bar. ^*^p < 0.05.

To investigate the neural development defects caused by ZIKV infection, a ZIKV-infected fetal mouse brain model was established. ICR mice were mated at embryonic day 0 (E0) and monitored for pregnancy. ZIKV virus stock was injected into one side of the cerebroventricular space of fetal brains at embryonic day 13.5 (E13.5) and sacrificed at 5 days post-injection (E18.5) (Figure 6A). The following results were observed: (1) The weight of the ZIKV-infected fetal bodies was significantly lower than the mock-infected littermates (Figure 6B). (2) The size of ZIKV-infected fetal brains was smaller compared to the mock-infected ones (Figure 6C). (3) The weight of ZIKV-infected fetal brains was significantly lower than the mock-infected ones (Figure 6D). Taken together, these data indicate that developmental defects are associated with ZIKV infection.

### ZIKV infection decreases DCX level and thickness of embryonic mouse brain cortex

**Figure 7:**
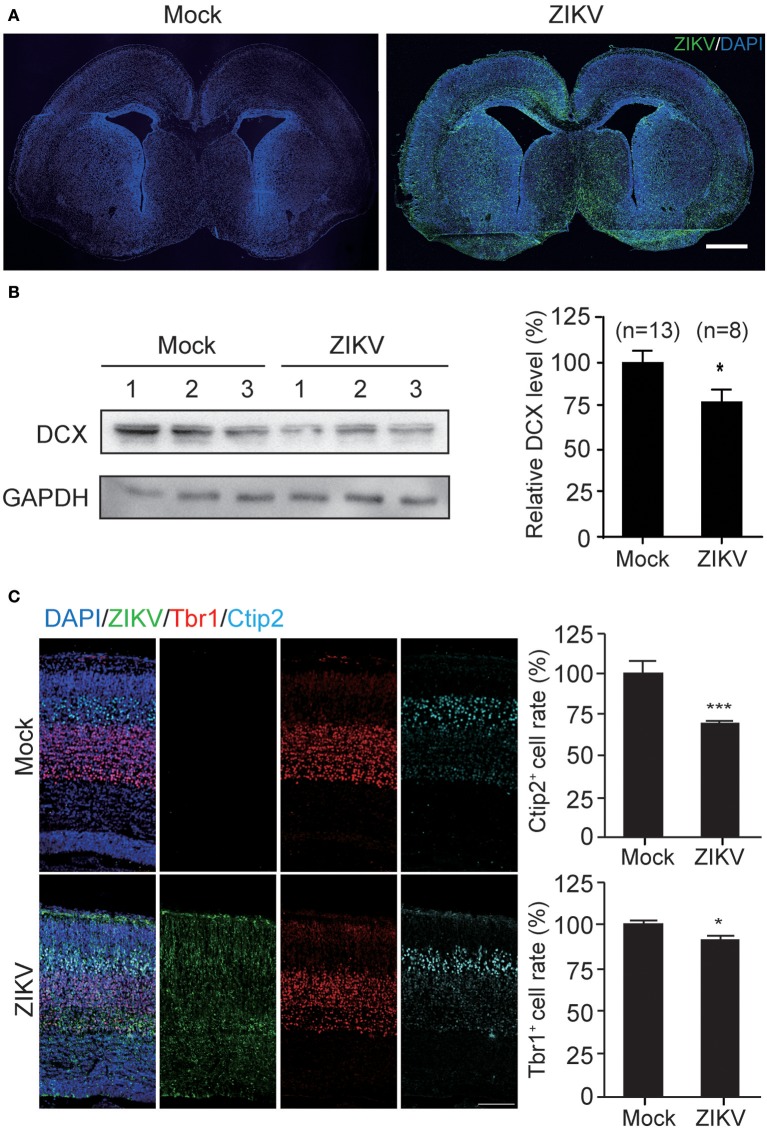
ZIKV infection decreases the expression of DCX in mouse fetal brain and causes defects in cortical layer structure. **(A)** ZIKV infection in mouse fetal brain. Infected fetal brains (E18.5) were perfused. Coronal sections of mock- and ZIKV-infected whole brain were stained with ZIKV serum and DAPI. Scale bar, 100 μm. **(B)** Protein level of DCX. Cortical protein samples from mock- and ZIKV-infected fetal littermates in one pregnant mouse were harvested. DCX protein in infected fetal brain samples was determined by immunoblotting assay. The DCX levels were quantified by densitometric analysis using the ImageJ software package by comparing to the mock-infected samples from at least 3 independent immunoblotting results. The n for each group is indicated below the column. ^*^p < 0.05. **(C)** Immunofluorescence images of cortical regions. Infected fetal brains (E18.5) were perfused. Coronal sections were stained with ZIKV antiserum (green), Tbr1 antibody (red), Ctip2 antibody (cyan) and DAPI to label nuclei (blue). Scale bar, 50 μm. The Ctip2+ and Tbr1+ cell rates were quantified. ^*^p < 0.05. ^***^p < 0.0001.

The infection of ZIKV in mouse fetal brain was detected with purified human antiserum by IFA. ZIKV infection signal was scattered throughout the whole fetal brains (Figure 7A), indicating efficient virus infection, virus replication and virus spread within the fetal brain. Additionally, the occurrence of ZIKV infections in different regions of the fetal brain suggests that ZIKV may have no obvious cell type-specific infection pattern, especially at the embryonic stage of fetal brain development. ZIKV signals were also detected in the ventricular zone/subventricular zone (VZ/SVZ) region which contains a large number of NPCs, indicating that mouse NPCs can also be infected by ZIKV.

To further determine the DCX protein level in these fetal brains, the cortical plates of ZIKV- or mock-infected fetal brains were obtained without PFA perfusion and fixation. Brain tissues were homogenized and lysed for protein extraction. Protein samples from three independent experiments were analyzed. The DCX protein levels of three ZIKV-infected fetuses and three mock-infected fetuses from the same pregnant mouse are shown as representatives (Figure 7B, left panel). The DCX levels were quantified densitometrically and normalized to GAPDH levels. The relative DCX protein levels decreased ~25% in ZIKV-infected fetal brains compared to the mock-infected ones (Figure 7B, right panel), suggesting that ZIKV infection can decrease DCX protein level in fetal brain.

DCX is important for neuronal migration, which determines cortex structure. To confirm whether the structures of the cortical plate is impaired due to DCX downregulation by ZIKV infection, mock- or ZIKV-infected fetal brain were examined by detecting different cortical layer markers: Tbr1 (specific for cortex layer VI); and Ctip2 (specific for cortex layer V) at E18.5 (Figure 7C, left panels). The Ctip2^+^ cell rates and Tbr1^+^ cell rates in ZIKV-infected fetal brains displayed about 30 and 10% reductions compared to the same region in the mock-infected ones, respectively (Figure 7C, right panels), indicating the cortical layers of ZIKV-infected fetal brains were thinner than the mock-infected ones.

### NS4A and NS5 downregulate DCX mRNA and protein levels

**Figure 8:**
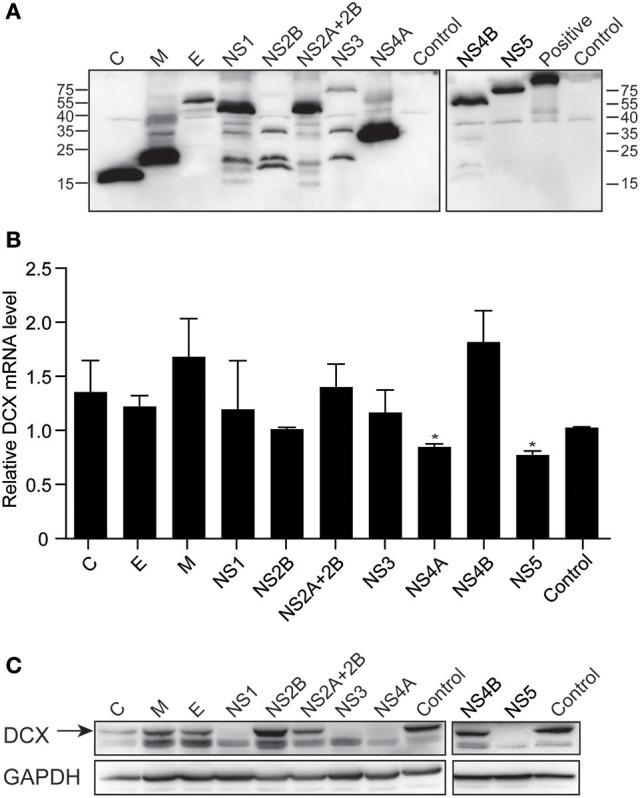
ZIKV NS4A and NS5 are involved in regulation of DCX mRNA and protein levels in NPCs. **(A)** Viral proteins expression. Cell lysates from lentivirus-transduced NPCs expressing ZIKV C, M, E, NS1, NS2B, NS2A+2B, NS3, NS4A, NS4B, and NS5 were prepared. FLAG-tagged viral proteins were detected by immunoblotting assay using anti-FLAG antibody. A FLAG-tagged beta-catenin protein (92 kDa) was using as positive control. **(B)** DCX mRNA level. NPCs were transduced with lentiviruses to express ZIKV viral proteins. Total RNA was extracted, reverse-transcribed and mRNA levels were assessed by qRT-PCR. Relative DCX mRNA levels were normalized to GAPDH mRNA levels in each sample and indicated as fold changes relative to control. ^*^p < 0.05. **(C)** DCX protein level. Cell lysates from lentivirus-transduced NPCs were prepared. DCX protein levels were determined by immunoblotting assay. DCX band was indicated by an arrow.

To determine which viral proteins are involved in the downregulation of DCX at both mRNA and protein levels, NPCs were transduced with lentiviruses to express the ten viral proteins, which were confirmed by immunoblotting against FLAG-tag (Figure 8A). The mRNA level of DCX was determined by qRT-PCR at 4 dpt. The DCX mRNA level was downregulated by NS4A and NS5, but not by other viral proteins (Figure 8B). Although the DCX protein level was downregulated by the C, NS1, NS3, NS4A, and NS5 proteins, the downregulation produced by NS4A and NS5 was more obvious effect (Figure 8C). Collectively, these data indicated that expression of NS4A and NS5 could downregulate both the DCX mRNA and protein levels.

## Discussion

There has been considerable effort to try and determine the molecular mechanisms responsible for the fetal brain malformations caused by ZIKV infections that were associated with the ZIKV outbreak in South and Central America that has been ongoing for the past 2 years (Musso and Gubler, 2016; Petersen et al., 2016). Recent studies demonstrated that ZIKV infection impairs the growth of induced pluripotent stem cells-derived NPCs, neurospheres and brain organoids (Garcez et al., 2016), and causes thinner cortex and smaller brain size for the fetal brain in mouse models (Li C. et al., 2016; Tang et al., 2016), highlighting the impact of ZIKV on NPC cell fate and neurogenesis. These studies indicate that cell apoptosis, autophagy, impaired proliferation and mitosis of NPC induced by ZIKV infection may contribute to ZIKV neuropathology (Huang et al., 2016; Liang et al., 2016; Li H. et al., 2016).

In this study, we utilized primary human NPCs, a clinic relevant cell type, to investigate the molecular mechanisms of how ZIKV induces fetal brain malformations. By performing a proteomic analysis, we found that the differentially expressed cellular proteins in NPCs during ZIKV infection were enriched in neural differentiation and migration processes, such as DCX, Reelin and FoxG1. Our data further verified that one of the key factors in neural migration, DCX, was downregulated during ZIKV infection in infected NPCs and fetal mouse brains. Additionally, two non-structural proteins of ZIKV, NS4A, and NS5, were shown to be involved in the downregulation of DCX at both the mRNA and protein levels. Interestingly, besides the defects in cell proliferation and viability of NPC during ZIKV infection, we found that the neural differentiation and migration process of NPC was affected by ZIKV infection, implying that DCX downregulation may be one of the potential mechanisms of ZIKV affecting neural development.

A recent report of proteomic analysis on ZIKV-infected mosquito cells highlighted the cellular responses to ZIKV infection including immune responses and signaling responses (Xin et al., 2017). In this study, ZIKV-infected NPCs were used for proteomic analysis, which helps to clarify how ZIKV infection affects NPC cell fate. The data revealed that ZIKV infection strongly alters multiple signaling pathways involved in determining NPC cell fate, such as the PI3K-AKT and p53 signaling pathways which were also shown to be affected in mosquito cells by proteomic analysis (Xin et al., 2017). The PI3K-AKT signaling pathway has been reported to regulate the proliferation and differentiation of NPCs (Peltier et al., 2007; Le Belle et al., 2011). The p53 signaling pathway is involved in regulating NPC renewal and differentiation (Yang et al., 2004; Zheng et al., 2008). This implies that the alteration of these two pathways by ZIKV infection may be potential molecular mechanisms for ZIKV neuro-pathogenesis. Furthermore, recent studies showed that ZIKV infection alters the AKT-mTOR signaling pathway in NPCs, which is another key cellular pathway in brain development, and also implicated a role for ZIKV NS4A, and NS4B (Liang et al., 2016). Given that the AKT-mTOR pathway is downstream of the PI3K-AKT-mTOR signaling axis, it seems that the alterations in the PI3K-AKT pathway as a consequence of ZIKV infection can be an alternative mechanism for the regulation of AKT-mTOR signaling pathway. Additionally, the observed perturbations to proteins within the p53 signaling pathway confirms a previous finding of increased p53 levels in ZIKV-infected NPCs (Ghouzzi et al., 2016).

Recent studies indicate that apoptosis and defects in cell proliferation caused by ZIKV contributes to the brain malformations. In this study, cellular proteins DCX, FoxG1 and Reelin were demonstrated to be differentially regulated. These proteins are involved in neural migration to generate attractive or repulsive cues, and form interactions between cell surface receptors and ligands to organize cytoskeletal elements in neural migration (Hatten, 2002). Our data also confirms a recent study, which reported that DCX was downregulated in primary strains of hNSCs infected with ZIKV (McGrath et al., 2017). DCX, a key factor for radial migration of neuroblasts and immature neurons in the cortex, is a X-linked gene highly related to lissencephaly and abnormal neuronal positioning (des Portes et al., 1998; Pilz et al., 1998). The loss of DCX in a rat model displays a defect in radial migration in the neocortex, suggesting an important role in cortex formation (Bai et al., 2003). Similarly, our data also demonstrated a decrease of DCX levels in ZIKV-infected fetal brain concomitant with an obvious defect in cortical structure formation, implying a potential mechanism for ZIKV-induced microcephaly via regulation of the neural migration process.

Interestingly, DCX is also modulated by human cytomegalovirus (HCMV), a leading cause of neural development disorders. Both the DCX mRNA and protein levels are strongly downregulated in NPCs infected with HCMV (Luo et al., 2010). These observations are similar to the findings in this study that DCX level was downregulated in both the ZIKV-infected NPC and mouse fetal brains. Although ZIKV and HCMV belong to different virus families and have distinct characteristics in their life cycles, both ZIKV and HCMV can be vertically transmitted in human and cause severe neuro-developmental defects in newborns. Although the causal link between ZIKV-mediated downregulation of DCX and microcephaly is not clear and other cellular factors may be involved, the fact that these two pathogens are able to downregulate DCX level in a similar fashion implies they may share some potential mechanisms associated with birth defects.

The downregulation of both DCX mRNA and protein levels by ZIKV infection implies the existence of distinct mechanisms in ZIKV that impact transcriptional and protein levels in the host. In this study, the expression of several viral proteins was correlated with the downregulation of DCX levels, and NS4A and NS5 were the two correlated with the downregulation on both mRNA level and protein level, and were closely related to the effect observed in ZIKV-infected NPCs. ZIKV viral protein NS4A has been shown to be involved in inhibiting the AKT-mTOR signaling pathway to induce autophagy in human NSCs (Liang et al., 2016). ZIKV NS4A has also been shown to be linked to translational repression of host cells (Hou et al., 2017), which may be related to the reduction of DCX protein level in NPCs. ZIKV NS5 protein targets and binds to human STAT2 for degradation to antagonize type I IFN (Grant et al., 2016), which could intensively impact multiple signaling pathways in NPCs. It is possible that NS5 may further affect NPC cell fate including DCX expression level. Our data extend the understanding of these two viral proteins in affecting cell fate of human NPCs. Further studies will be required to elucidate the specific means by which ZIKV NS4A, and NS5 influence the expression level of DCX.

## Author contributions

M-HL, Y-PT, and HY conceived and designed the research. XJ, XD, and Y-PZ performed the experiments. S-HL, GG, and H-MX offered advices and technical assistance. XJ and XD analyzed the data. XJ and SR wrote the paper. M-HL approved the manuscript after careful analysis.

### Conflict of interest statement

The authors declare that the research was conducted in the absence of any commercial or financial relationships that could be construed as a potential conflict of interest.
